# Medical Witness

**Published:** 1905-11

**Authors:** D. L. Field

**Affiliations:** Jeffersonville, Ind.


### MEDICAL WITNESS.

BY D. L. FIELD, M.D., JEFFERSONVILLE, IND.

There can be no more trying predicament for a sensitive, conscientious physician than when on the witness stand in a courtroom.

How liable to be confused; to say what he really does not want to say; and when he is discharged, feel a misgiving as to whether he has enlightened the court and jury, and a feeling of humiliation, and an apprehension that under the merciless cross-examination of the lawyer, he has made an ass of himself! He is the victim of stage fright.

Let him be given the same categorical, hypothetical questions in writing, to be answered in writing in the privacy of the doctor's office, and he will, no doubt, acquit himself creditably, and better enlighten the court. But put the same questions to him in a crowded court-room, with the lawyers glaring at him, and the jury scrutinizing him, and unless he is very level-headed, he will amount to almost nothing as an expert witness.

To be an expert medical witness involves the usefulness and respectability of the profession. The lives, property, liberty and honor of individuals may depend upon the decision of medical experts; therefore, they can not be too cautious and conscientious in giving an opinion for the guidance of the court.

For example, a will is presented for probate, and the issue is the competency of the testator, is raised as to whether he was of sane and disposing mind at the time the will was made; the delicate and responsible opinion of the expert will have much to do with determining the issue in the minds of the jury.

Here comes in that *hard* problem—to decide upon hypothetical questions, whether the party was sane or insane at the time, or before the will was made. Here the medical witness will often, if his opinion be well considered and intelligently delivered, form the basis of a verdict. His responsibility is grave, and he can not exercise too much care in giving an opinion.

A person in active business is noticed to assume a new character; to adopt new habits in action and speech. Friends regard him of unsound mind, and seek a judicial inquiry to determine the fact. A medical opinion is almost invariably required in order to form a well-founded adjudication in the case. The business and property interests involved in a decision render the medical testimony highly important, and a medical man must keep his eyes skinned for fear in giving his opinion he may not become a party to a conspiracy to wrongfully dispossess a sane man of his property and his liberty.

Again, an individual may become the victim of an insidious form of mental unsoundness which creeps on him so gradually that he may squander a hard-earned estate by wild ventures, or become the prey of wily, designing, dishonest vampires, who will suck the financial life-blood from him, and work the robbery of those dependent upon him ; and it demands an astute, alert expert to protect, by his skill and knowledge, the weak and deserving, while at the same time thwarting the schemes of rascals.

In actions of divorce, involving the delicate relations of husband and wife, and consequently the capacity of the parties to sustain that relation, and where is brought in question such acts as amount to a violation of the marital rights or duties of the parties, the opinion of the physician often forms the basis of a decision. The same is true of most cases involving legitimacy.

In cases of malpractice, as a ground for a claim for damages, the testimony of an expert will usually determine the case.

It is, however, in criminal cases that the medical evidence has attracted the largest share of public attention, and of public approbation or condemnation. Here, as in civil cases, it has a double character. It embraces facts from which opinions and judgments are formed. Skilled investigation becomes necessary in most cases of homicide, rape, infanticide and abortion. In such cases, when the expert is employed, it is because he is supposed to possess the skill and knowledge to find out the truth in any given case. He is almost indispensable in coroners' investigations, and surely so in post mortems. And right here let me say that, if possible, every coroner should be a physician.

In hypothetical examinations of a doctor, he becomes almost lawyer, judge and jury. He should, however, be the simple teller of the truth, and never stop to consider what may be the effect of his opinion honestly given. Because he may be summoned on one side is no reason why he should lean in his opinions to one side ; but let him stick to "the truth, the whole truth, and nothing but the truth," whether it fits the purpose of the side for whom he was summoned, or *not.* Medical witnesses should never become partisan or partial. Answer all questions put to you, honestly, simply, directly, intelligently, and, *above all,* for mercy sake, don't assume an all-wise, pompous air. Never assume to know it all, or pretend to be profoundly learned ; if you *do,* you will be pretty sure to come to grief before you are done.

